# Evaluation of Bioactive Compounds, Pharmaceutical Quality, and Anticancer Activity of Curry Leaf (*Murraya koenigii* L.)

**DOI:** 10.1155/2014/873803

**Published:** 2014-02-16

**Authors:** Ali Ghasemzadeh, Hawa Z. E. Jaafar, Asmah Rahmat, Thiyagu Devarajan

**Affiliations:** ^1^Department of Crop Science, Faculty of Agriculture, University Putra Malaysia, 43400 Serdang, Selangor, Malaysia; ^2^Department of Nutrition & Dietetics, Faculty of Medicine & Health Sciences, University Putra Malaysia (UPM), 43400 Serdang, Selangor, Malaysia; ^3^Malaysian Agriculture Research and Development Institute, 43400 Serdang, Selangor, Malaysia

## Abstract

In this study, we investigated some bioactive compounds and pharmaceutical qualities of curry leaf (*Murraya koenigii* L.) extracts from three different locations in Malaysia. The highest TF and total phenolic (TP) contents were observed in the extracts from Kelantan (3.771 and 14.371 mg/g DW), followed by Selangor (3.146 and 12.272 mg/g DW) and Johor (2.801 and 12.02 mg/g DW), respectively. High quercetin (0.350 mg/g DW), catechin (0.325 mg/g DW), epicatechin (0.678 mg/g DW), naringin (0.203 mg/g DW), and myricetin (0.703 mg/g DW) levels were observed in the extracts from Kelantan, while the highest rutin content (0.082 mg/g DW) was detected in the leaves from Selangor. The curry leaf extract from Kelantan exhibited higher concentration of gallic acid (0.933 mg/g DW) than that from Selangor (0.904 mg/g DW) and Johor (0.813 mg/g DW). Among the studied samples, the ones from Kelantan exhibited the highest radical scavenging activity (DPPH, 66.41%) and ferric reduction activity potential (FRAP, 644.25 **μ**m of Fe(II)/g) followed by those from Selangor (60.237% and 598.37 **μ**m of Fe(II)/g) and Johor (50.76% and 563.42 **μ**m of Fe(II)/g), respectively. A preliminary screening showed that the curry leaf extracts from all the locations exhibited significant anticarcinogenic effects inhibiting the growth of breast cancer cell line (MDA-MB-231) and maximum inhibition of MDA-MB-231 cell was observed with the curry leaf extract from Kelantan. Based on these results, it is concluded that Malaysian curry leaf collected from the North (Kelantan) might be potential source of potent natural antioxidant and beneficial chemopreventive agents.

## 1. Introduction

Plant-derived substances have recently attracted great interest, owing to their versatile applications. Medicinal plants are the richest source of bioactive compounds used in traditional and modern medicine as nutraceuticals and food supplements, pharmaceutical intermediates, and chemical entities for synthetic drugs [1]. Flavonoids are an important group of plant secondary metabolites with a wide range of biological activities [2]. These natural products have been shown to have antioxidant properties and are capable of scavenging free superoxide radicals, thus providing antiaging benefits as well as reducing the risk of cancer. Park et al. [3] showed that some flavonoid components in green tea were effective in inhibiting cancer by inducing the mechanisms that cause cancer cell death and inhibit tumor invasion. It has been found that flavonoids reduce the lipid and glucose levels in blood and support the human immune system [4, 5]. The health-promoting effects of flavonoids are the result of their ability to induce the activity of protective enzyme systems [6]. Breast cancer is the most common cancer in women and men, diagnosed in more than 1.2 million women worldwide and accounting for approximately 23% of all cancers reported. This cancer was reported as a most common form of cancers among Malaysian women since 2003 (18% of all cancers cases). A woman in Malaysia had a 1 in 20 chance of developing breast cancer in her lifetime [7]. Several studies have suggested that flavonoids such as catechin and quercetin are able to control the growth of cancer cells in human body [8, 9]. The plant *Murraya koenigii* (L.) Spreng, commonly known as curry leaf or Pokok kari (Daun kari) in Malaysia, belongs to the family of Rutaceae, which is native to India and now distributed in most of Southern and Southeast Asia [10]. Curry leaf has a slightly pungent, bitter, and feebly acidulous taste and is widely used for cooking in Malaysia and other Asian countries. It is also one of the traditional folk remedies that contains several interesting bioactive compounds with health-promoting properties [10]. Different parts of *M. koenigii* have been used in traditional Ayurveda medicine for the treatment of cough, hypertension, hysteria, hepatitis, rheumatism, poisonous bites, and skin eruptions. In addition, curry leaf has been reported to have antitumor [11], antioxidant [12], anti-inflammatory [13], antihyperglycemic [14], and hypoglycemic effects [15]. Nevertheless, the information about the secondary metabolite content and the antioxidant and anticancer activity of Malaysian curry leaf is still scarce. Additional research in this direction would help to further elucidate health benefits provided by this functional food additive.

The present investigation was undertaken to determine the bioactive compounds and antioxidant activity in the leaf extracts of *M. koenigii* collected from three different locations in Malaysia. In addition, anticancer properties of the curry leaf extracts have been investigated in vitro by using a breast cancer cell line.

## 2. Materials and Methods

### 2.1. Plant Material

Fresh leaves of curry leaf were collected locally from three different province of Malaysia, namely, Kelantan (Bachok, North), Selangor (BFK, Central), and Johor (Nasuha, South). The samples were identified by Malaysian Agriculture Research and Development Institute (MARDI). Voucher specimens of *M. koenigii* Kelantan (MTM0018/1), Selangor (MTM0018/3), and Johor (MTM0018/2) were collected. Malaysian Agriculture Research and Development Institute (MARDI) verified and kept samples. The leaves were shade-dried and were powdered using mechanical grinder. These powered materials were used for further analysis.

### 2.2. Preparation of Extract

Leaf samples (0.25 g) were extracted with 20 mL of methanol on a shaker for 2 h at room temperature. The extract solution was treated with 5 mL of hydrochloric acid (6 M) and refluxed for 2 h at 90°C. The hydrolysed samples were cooled to room temperature and filtered through a 0.45 *μ*m membrane [16].

### 2.3. Determination of Total Phenolic Content

The content of total phenolics from the curry leaf was evaluated by the Folin-Ciocalteu method [17]. Briefly, 1 mL of extract was added to deionized water (10 mL) and Folin-Ciocalteu phenol reagents (1.0 mL). After 5 min, 20% sodium carbonate (2.0 mL) was added to the mixture. The solution was kept in total darkness, and the absorbance was measured at 750 nm using a spectrophotometer (U-2001, Hitachi Instruments Inc., Tokyo, Japan). Gallic acid was used for the calibration curve.

### 2.4. Determination of Total Flavonoids

The total flavonoid content of the curry leaf extract was determined using a modified colourimetric method [18]. Briefly, extracts of each plant material (1 mL) were diluted with 4 mL water in a 10 mL volumetric flask. Initially, 5% NaNO_2_ solution (0.3 mL) was added to each volumetric flask; after 5 min, 10% AlCl_3_ (w/v) was added; and at 6 min, 1.0 M NaOH (2 mL) was added. Absorbance of the reaction mixture was read at 430 nm. Quercetin was used for the calibration curve.

### 2.5. Separation and Analysis of Flavonoids and Phenolic Acids by HPLC

Reversed-phase HPLC was used to assay flavonoid composition. The Agilent HPLC system used consisted of a Model 1100 pump equipped with a multisolvent delivery system, an L-7400 (Hitachi) ultraviolet (UV) detector, and fitted with an Agilent C18 (5 *μ*m, 4.6 × 250 mm) column. The mobile phase consisted of (A) 0.03 M phosphoric acid in water and (B) methanol. The mobile phase was filtered under vacuum through a 0.45 um membrane filter before use. The flow rate was maintained at 1 mL/min and UV absorbance was measured at 260–360 nm. The operating temperature was maintained at room temperature [19, 20]. Identification of the compounds was achieved by comparison of retention times with standards, UV spectra, and UV absorbance ratios after coinjection of samples and standards.

### 2.6. Determination of Antioxidant Activity

#### 2.6.1. Ferric Reducing Antioxidant Potential (FRAP) Assay

The stock solutions consisted of 300 mM acetate buffer, 10 mM TPTZ (2,4,6-tripyridyl-S-triazine) solution in 40 mM HCl, and 20 mM FeCl_3_ solution. Acetate buffer (25 mL) and TPTZ (2.5 mL) were mixed, and 2.5 mL FeCl_3_ was added. Leaf extract (150 *μ*L) was added to 2850 *μ*L of the FRAP solution and kept for 30 min in the dark place. The absorbance of solution was measured at 593 nm using a spectrophotometer (U-2001, Hitachi Instruments Inc., Tokyo, Japan) [21].

#### 2.6.2. 1,1-Diphenyl-2-picrylhydrazyl (DPPH) Assay

The radical scavenging ability was determined using the method described by Mensor et al. [22]. Briefly, an alcohol solution of DPPH (1 mL, 3 mg/mL) was added to 2.5 mL samples containing different concentrations of extracts. The samples were first kept in the dark at room temperature and their absorbance was read at 518 nm after 30 min. The antiradical activity was determined using the following formula:

(1) Percent (%)inhibition  of  DPPH  activity  =[(A0−A1)A0 ]×100%,

where *A*_0_ is the absorbance value of the blank sample or control reaction and *A*_1_ is the absorbance value of the test sample. The optic densities of the samples and controls were measured in comparison to ethanol. BHT (butylhydroxytoluene) and *α*-tocopherol were used as positive controls.

### 2.7. Determination of Anticancer Activity

#### 2.7.1. Cell Culture and Treatment

Human breast carcinoma cell lines (MDA-MB-231) and normal human mammary epithelial cell (MCF-10A) were cultured in 100 *μ*L of RPMI 1640 media (Roswell Park Memorial Institute) containing 10% fetal bovine serum (FBS). Cell lines were incubated overnight at 37°C in 5% CO_2_ for cell attachment.

#### 2.7.2. MTT (3-(4,5-Dimethylthiazol-2-yl)-2,5-diphenyltetrazolium Bromide) Assay

The assay was conducted as follows: cancer cells were seeded in 96-well plates at a density of 1 × 10^4^ cells/well in 100 *μ*L RPMI. At 24 h after seeding, the medium was removed and the cells were incubated for 3 days with RPMI in the absence or presence of various concentrations of curry leaf extracts. Extracts concentrations used ranged between 20, 40, 80, 160, 320, and 640 *μ*g/mL. After incubation, 20 *μ*L of MTT reagent was added into each well. The plate was incubated again in a CO_2_ incubator at 37°C for 4 h. The resulting MTT-products were determined by measuring the absorbance at 570 nm using ELISA reader [23]. Each point represents the mean of triplicate experiments. The cell viability was determined using the formula

(2) $$\begin{array}{c} \text{Viability}\;\left(\%\right)=\frac{\text{optical}\;\text{density}\;\text{of}\;\text{sample}}{\text{optical}\;\text{density}\;\text{of}\;\text{control}}\times 100. \end{array}$$

### 2.8. Statistical Analysis

All analytical values shown represent the means of three replicates. Data were analysed using analysis of variance by Statistical Analysis System (SAS 9.0). Mean separation test between treatments was performed using Duncan multiple range test and a *P*  value ≤ 0.05 was regarded as significant.

## 3. Results and Discussion

### 3.1. Total Flavonoid Content and Composition

The results obtained from a preliminary analysis of flavonoid compounds are shown in Table 1. There was a significant difference in the total flavonoid (TF) content among the extract of curry leaf originated from different locations. The highest TF content was observed in the curry leaf from Kelantan (3.771 ± 0.546 mg/g DW) followed by that from Selangor (3.146 ± 0.524 mg/g DW) and Johor (2.801 ± 0.493 mg/g DW). In this study, six flavonoid compounds were identified in curry leaf extracts. The highest rutin concentration was observed in the plants from Selangor (0.082 ± 0.011 mg/g DW). Zhang et al. [24] identified rutin in the concentration of 0.356 mg/100 g DW in the curry leaf from Florida, USA. The samples from Kelantan showed a high quercetin content (0.350 ± 0.024 mg/g DW), but there was no significant location-specific difference in this parameter. As shown in Table 1, epicatechin was detected in the plants from two locations, Kelantan and Selangor, with the highest level observed in those from Kelantan (0.678 ± 0.032 mg/g DW).

**Figure 1:**
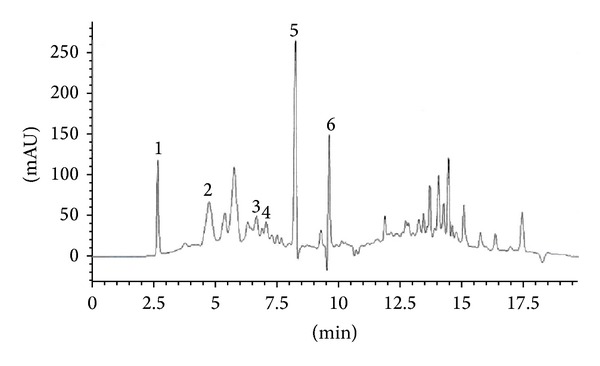
HPLC chromatogram of curry leaf extracts, Kelantan location. Identification of compounds: catechin (1), epicatechin (2), rutin (3), naringin (4), myricetin (5), and quercetin (6).

Contrary to epicatechin, catechin was detected in the curry leaf from all locations and the highest catechin concentration was observed in the curry leaf extract from Kelantan (0.325 ± 0.057 mg/g DW). Naringin was detected only in the Kelantan extract (0.203 ± 0.036 mg/g DW). Myricetin is a flavonoid found in many herbs, fruits, and vegetables as well as other plants [25]. Myricetin exerts powerful biological effects including anticancer and antioxidant activities [26, 27]. The results showed that myricetin was the most abundant flavonoid in curry leaf and a high myricetin concentration was detected in the samples from Kelantan (0.703 ± 0.063 mg/g DW) followed by those from Selangor (0.600 ± 0.078 mg/g DW) and Johor (0.502 ± 0.040 mg/g DW).  Figure 1 shows the HPLC chromatogram of curry leaf extracts from Kelantan location.

### 3.2. Total Phenolic Content and Identification of Phenolic Acids

Phenolics that exhibit antioxidant activity are known to be mainly flavonoids and phenolic acids. Phenolic acids are the major class of phenolic compounds widely occurring in the plant kingdom, especially in herbs and vegetables. As shown in Table 2, the curry leaf from Kelantan had the highest total phenolic (TP) content (14.371 ± 0.654 mg/g DW) followed by that from Selangor (12.272 ± 0.541 mg/g DW) and Johor (12.020 ± 0.391 mg/g DW). A significant difference (*P* ≤ 0.05) was observed between Kelantan and Selangor, while the difference between Selangor and Johor was insignificant. Wong et al. [28] investigated the TP content in 25 tropical plants and reported that curry leaf was the second after petai beans (26.4 mg/g DW) in regard to TP concentration. However, compared to some other medicinal herbs such as *Melisa officinalis* (13.2 mg/g DW), *Taraxacum officinale* (12.6 mg/g DW), *Acorus calamus* (12.45 mg/g DW), *Echinacea purpurea* (15.1 mg/g DW), *Syzygium aromaticum* (8.96 mg/g DW), and *Salvia officinalis* (8.25 mg/g DW), curry leaf exhibited a substantial TP content (12.02–14.37 mg/g DW) [29]. It is evident that the TP content measured by the Folin-Ciocalteu method does not reveal quality or quantity of the individual phenolic compounds present in plant extracts [30]. In the current study, three phenolic acids including gallic acid, cinnamic acid, and ferulic acid were identified in the curry leaf extracts from three locations. The curry leaf extract from Kelantan exhibited the highest concentration of gallic acid (0.933 ± 0.076 mg/g DW) compared to that from Selangor (0.904 ± 0.40 mg/g DW) and Johor (0.813 ± 0.065 mg/g DW). In addition, no significant difference was observed in the concentration of gallic acid between Kelantan and Selangor plants.

As Table 2 shows, cinnamic acid was not detected in the curry leaf from Kelantan, while the plants from Selangor exhibited a higher cinnamic acid content (0.077 ± 0.021 mg/g DW) than those from Johor (0.068 ± 0.019 mg/g DW). Ferulic acid in high concentrations was shown to inhibit photoperoxidation of linoleic acid [31]. The most interesting finding here was that ferulic acid could be detected only in the curry leaf extracts from Kelantan, which has not been previously reported. Vanillic acid was detected in curry leaf extracts from all locations and extracts from Kelantan exhibited the highest concentration of vanillic acid (0.788 ± 0.103 mg/g DW) compared to Selangor (0.659 ± 0.058) and Johor (0.527 ± 0.111).

### 3.3. Antioxidant Activity

#### 3.3.1. Ferric Reducing Antioxidant Potential (FRAP) Assay

**Figure 2:**
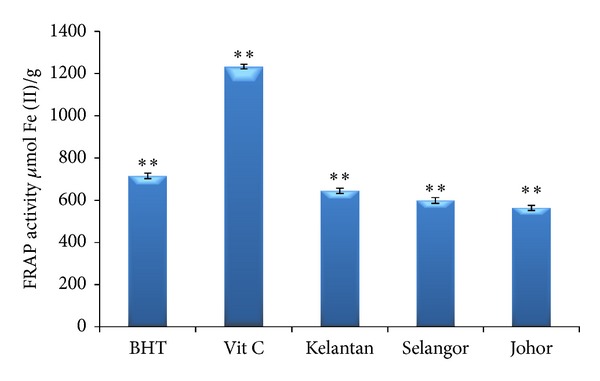
FRAP activity of curry leaf collected from three different locations compared with positive controls' butylated hydroxytoluene (BHT) and vitamin C. Bars represent standard error of means. **represents significance at *P* < 0.01.

The FRAP assay is based on the reduction of ferric tripyridyltriazine (Fe(III)-TPTZ) to ferrous tripyridyltriazine (Fe(II)-TPTZ) at low pH. The FRAP assay has been widely used to estimate the antioxidant content/power of dietary polyphenols [32]. As Figure 2 shows, the reducing power in the curry leaf extracts was in the range between 644.25 (Kelantan) and 563.42 *μ*m of Fe(II)/g (Johor). The FRAP values for all three curry leaf extracts were significantly lower than those shown by the standard antioxidants, butylated hydroxytoluene (BHT) and vitamin C (715.1 and 1232.24 *μ*mol Fe (II)/g, resp.). In this study, we used the FRAP assay because it is quick and simple in measuring the antioxidant capacity of different products including plants, wines, and animal tissues [33]. Many previous studies highlighted the potential role of flavonoids and phenolic acids of herbs and spices which may act as antioxidants [2, 5, 9, 22, 26, 29]. In general, the antioxidant activity of flavonoids is defined by the substitution pattern and structure of hydroxyl groups. In the flavonoid chemical structure, the 3′,4′-orthodihydroxy configuration in the ring B and the 4-carbonyl group in the ring C define the radical scavenging activity. The presence of 3- and 5-OH groups provides a catechol-like structure in the ring C, which is also essential for the antioxidant activity of flavonoids [29]. Furthermore, the presence of the C_2_-C_3_ double bond conjugated with a 4-keto group is responsible for electron delocalization from the ring B and enhancement of the free radical scavenging activity. In the absence of the o-dihydroxy structure in the ring B, a catechol structure in the ring A can compensate for the flavonoid antioxidant activity [34].

#### 3.3.2. 1,1-Diphenyl-2-picrylhydrazyl (DPPH) Assay

**Figure 3:**
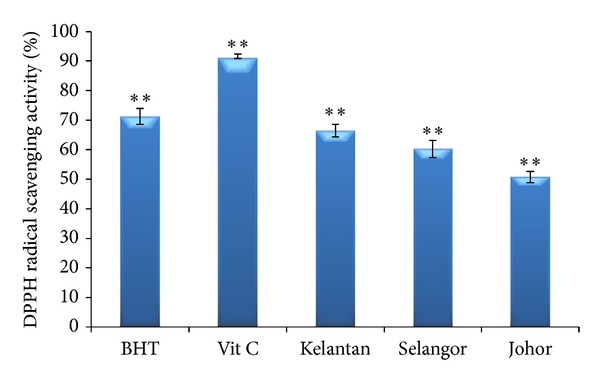
DPPH radical scavenging activity of curry leaf collected from three different locations compared with positive controls' butylated hydroxytoluene (BHT) and vitamin C. Bars represent standard error of means. **represents significance at *P* ≤ 0.01.

**Figure 4:**
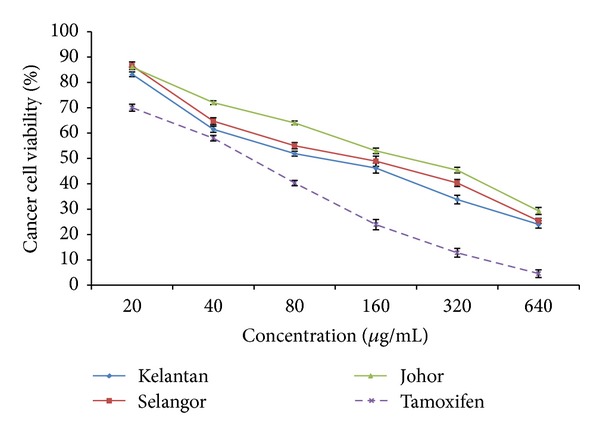
Dose-dependent anticancer of curry leaf extracts from 3 different locations (Kelantan, Selangor, and Johor) towards MDA-MB-231 cell line as determined by the MTT assay. Tamoxifen was used as a positive control. Bars represent standard error of means.

The DPPH radical scavenging by antioxidants is thought to be due to their hydrogen-donating ability. Among the studied locations, the curry leaf extracts from Kelantan exhibited the highest DPPH scavenging activity (66.41%) followed by those from Selangor (60.23%) and Johor (5.76%). In addition, a significant difference in the DPPH activity was observed among the three locations (Figure 3). The results of the current study showed that the DPPH radical scavenging of the curry leaf extracts was lower than that of BHT (83.7%) and vitamin C (92.3%) at 20 mg/mL. The IC_50_ (fifty percent free radical scavenging) values were 8.65, 10.4, and 10.88 mg/mL for Kelantan, Selangor, and Johor extracts, respectively. The DPPH scavenging activity observed in this study was comparable to that reported by Marinova et al. [35] but higher than that shown by Odukoya et al. [36]. Analysis of the data presented in Figures 3 and 4 indicates that in curry leaf extracts the FRAP activity did not markedly differ from the free radical scavenging activity measured by the DPPH assay because both of these assays work by the same mechanism (single-electron transfer). Antioxidant compounds such as polyphenols may be more efficient as ferric reducing agents than free radical scavenges because of steric hindrance [29].

Pulido et al. [37] reported that in most cases the ability of antioxidants to reduce the ferric ion is correlated with the antioxidant parameters assessed by other methods including the DPPH radical scavenging assay. Arnous et al. [38] reported a strong correlation between the DPPH free radical scavenging ability and ferric ion reduction in wines.

### 3.4. Correlations between TF, TP, FRAP, and DPPH Activity

Table 3 shows that the FRAP activity had a significant positive correlation with TP (*R* ^2^ = 0.92; *P* ≤ 0.05) and TF (*R* ^2^ = 0.88; *P* ≤ 0.05) suggesting that in curry leaf the increase in the FRAP activity might be due to the increase in the TP and TF. Furthermore, the DPPH scavenging activity in curry leaf extracts demonstrated a significant positive correlation with TP (*R* ^2^ = 0.85; *P* ≤ 0.05) and TF (*R* ^2^ = 0.91; *P* ≤ 0.05). An interesting finding (Table 3) is that the FRAP activity showed more correlation with TF than with TP, while the DPPH activity correlated more with TF than TP. A linear correlation between the TP content and antioxidant capacity has been demonstrated in some previous studies [39–41], while other found poor or no linear correlation between the total antioxidant activity and TP content [28, 42]. Our results confirm the importance of flavonoids and phenolics as the antioxidant agents in curry leaf extracts that significantly contribute to the total antioxidant capacity.

### 3.5. Anticancer Activity

A preliminary screening showed that curry leaf extracts from the three locations exhibited a significant anticancer activity against MDA-MB-231 cancer cells, with the inhibition rate of 67.2, 59.8, and 53.6% at concentration of 320 *μ*g/mL from Kelantan, Selangor, and Johor, respectively (Figure 4). MDA-MB-231 cells treated with tamoxifen (positive control) showed 87.2% inhibition at the same concentration. The IC_50_ values (fifty percent free radical scavenging) of the curry leaf extracts from Kelantan, Selangor, and Johor for MDA-MB-231 cells were 103.4, 149.6, and 194.3 *μ*g/mL, respectively. Flavonoids are among the most potent ingredients that underlie the protective effect of diets rich in fruits and vegetables with respect to colorectal cancer [42]. Hence, flavonoid compounds could probably be responsible for the anticancer activity of curry leaf. The American National Cancer Institute recommends considering crude herbal extracts that do not decrease the viability of normal cells below 76% [43] as safe for human consumption. In the current study, the highest flavonoid content and anticancer activity against MDA-MB-231 cells were demonstrated for the Kelantan curry leaf suggesting that the high anticancer activity in curry leaf extracts may be attributed to the high concentration of flavonoids such as myricetin, epicatechin, and quercetin known as potent anticancer agents. However, more research is needed for a better understanding of the association between these flavonoids and anticancer activity in curry leaf extracts.

This study produced results which corroborate the findings of a great deal of the previous work in this field. Mahenine, a carbazole alkaloid isolated from curry leaf, has been reported to induce apoptosis in human myeloid HL-60 cancer cells by downregulating cell survival factors and disrupting the cell cycle progression [44–46]. Antitumorogenic activity of a curry leaf extract (stem bark) against MCF-7 breast cancer cells has been reported by Handral et al. [47].

Previous studies were focused on different varieties and species of *Murraya koenigii* from different sampling areas like India, Indonesia, and so forth. Environmental conditions such as soil, sunlight, temperature, rainfall, storage conditions, altitude as well as different harvesting procedures, method of collection and time, and manufacturing processes such as drying, selecting, extracting, and purifying can generate substantial variability in bioactive compounds and plant [48, 49]. Information about pharmaceutical quality of Malaysian curry leaf from different collection locations is rare and such data from current study could be useful for future studies. Meanwhile, the results of current study have shown that production of secondary metabolites and pharmaceutical quality of curry leaf was influenced by grown area in Malaysia.

**Figure 5:**
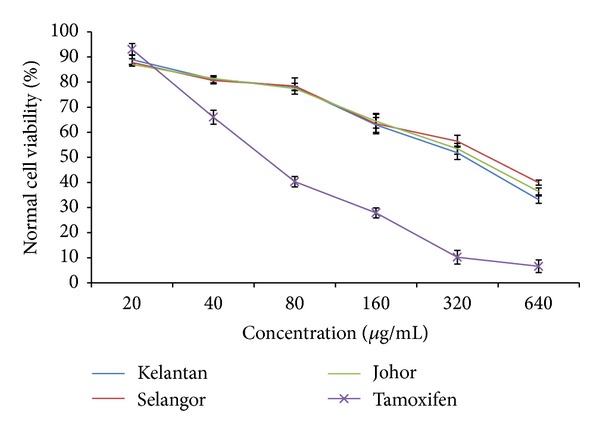
Effect of curry leaf extracts from three different locations (Kelantan, Selangor, and Johor) on normal cell viability (normal human mammary epithelial cell, MCF-10A.).

In a herbal supplement, one ingredient may provide the desired therapeutic benefits while others may have toxic effects for humans. Malaysian herbs and spices may also contain certain toxic components which are not well investigated. In this study, the curry leaf extracts were evaluated as nontoxic for normal (MCF-10A) cells as the IC_50_ values were greater than 320 *μ*g/mL (Figure 5). The viability of normal cells treated with the curry leaf extract from Kelantan at the IC_50_ (121.4 *μ*g/mL) was 71.6%. According to the obtained results, all the curry leaf extracts showed toxic effects at the concentrations above 320 *μ*g/mL: the IC_50_ values of Kelantan, Selangor, and Johor extracts for normal cells were 334.5, 352.8, and 377.2 *μ*g/mL, respectively. Paranagama et al. [50] reported that the IC_50_ value for the toxic effect of curry leaf was 240 *μ*g/mL.

## 4. Conclusions

The curry leaf with the highest TF and TP contents also showed the highest antioxidant activity as indicated by the FRAP and DPPH assays. Among the three studied locations, Kelantan plants which had high levels of phenolic acids (especially gallic acid) and flavonoids (especially myricetin, epicatechin, and quercetin) also exhibited a significant anticancer activity. Thus, the quantitative and qualitative analyses of major individual flavonoids and phenolics could provide an explanation for the correlation between the TP content and antioxidant capacity in curry leaf extracts. A wide concentration range of flavonoids and phenolic acids and variations in the antioxidant activity in curry leaf extracts could be due to several factors including growing location, altitude, climate, temperature, and diversity of natural vegetation in the area. Furthermore, the composition of phenolic acids and flavonoids and the related antioxidant activity can be useful for standardization of curry leaf extracts for further pharmaceutical applications. One of the more significant findings to emerge from this study is that the curry leaf extracts exhibited a promising anticancer activity on MDA-MB-231 human breast cancer cell line. The extracts contained substantial amounts of effective flavonoid compounds such as myricetin, epicatechin, and quercetin which showed potency in the growth inhibition of breast cancer cells. Subsequently, our MTT assay indicated that curry plants grown in the North of Malaysia (Kelantan) are a potential source of anticarcinogenic therapeutic compounds. More information on other bioactive components in curry leaf would help to further evaluate the anticancer activity of this medicinal plant.

## Figures and Tables

**Table 1:** The concentrations of TF and some flavonoid compounds detected from curry leaf in three locations.

	Kelantan	Selangor	Johor
TF	3.771 ± 0.546^a^	3.146 ± 0.524^b^	2.801 ± 0.493^c^
Rutin	0.042 ± 0.008^b^	0.082 ± 0.011^a^	0.048 ± 0.009^b^
Quercetin	0.350 ± 0.024^a^	0.341 ± 0.015^a^	0.305 ± 0.028^a^
Epicatechin	0.678 ± 0.032^a^	0.601 ± 0.050^a^	ND
Catechin	0.325 ± 0.057^a^	0.245 ± 0.044^b^	0.204 ± 0.032^b^
Naringin	0.203 ± 0.036^a^	ND	ND
Myricetin	0.703 ± 0.063^a^	0.600 ± 0.078^b^	0.502 ± 0.040^c^

All analyses are the mean of triplicate measurements ± standard deviation. Results expressed in mg/g DW. Means not sharing a common letter were significantly different at *P* ≤ 0.05.

**Table 2:** The concentrations of TP and some phenolic acids detected from curry leaf in three locations.

	Kelantan	Selangor	Johor
TP	14.371 ± 0.654^a^	12.272 ± 0.541^b^	12.020 ± 0.391^b^
Gallic acid	0.933 ± 0.076^a^	0.904 ± 0.040^a^	0.813 ± 0.065^b^
Cinnamic acid	ND	0.077 ± 0.021^a^	0.068 ± 0.019^a^
Ferulic acid	0.281 ± 0.055^a^	ND	ND
Vanillic acid	0.788 ± 0.103^a^	0.659 ± 0.058^a^	0.527 ± 0.111^a^

All analyses are the mean of triplicate measurements ± standard deviation. Results are expressed in mg/g DW. Means not sharing a common letter were significantly different at *P* ≤ 0.05.

**Table 3:** Correlation between TF, TP, and antioxidant activity in curry leaf extract.

		1	2	3	4
1	TF	1			
2	TP	0.89**	1		
3	FRAP	0.92**	0.88**	1	
4	DPPH	0.85**	0.91**	0.95**	1

**Represents significance at *P* ≤ 0.01.
